# Impact of apoptotic adipose-derived mesenchymal stem cells on attenuating organ damage and reducing mortality in Rat sepsis syndrome induced by cecal puncture and ligation

**DOI:** 10.1186/1479-5876-10-244

**Published:** 2012-12-07

**Authors:** Chia-Lo Chang, Steve Leu, Hsin-Ching Sung, Yen-Yi Zhen, Chung-Lung Cho, Angela Chen, Tzu-Hsien Tsai, Sheng-Ying Chung, Han-Tan Chai, Cheuk-Kwan Sun, Chia-Hung Yen, Hon-Kan Yip

**Affiliations:** 1 Division of Colorectal Surgery, Department of Surgery, Kaohsiung Chang Gung Memorial Hospital and Chang Gung University College of Medicine, Kaohsiung, Taiwan, Republic of China; 2 Center for Translational Research in Biomedical Sciences, Kaohsiung Chang Gung Memorial Hospital and Chang Gung University College of Medicine, Kaohsiung, Taiwan, Republic of China; 3 Department of Anatomy, Chang Gung University, Taoyuan, Taiwan, Republic of China; 4 Division of Cardiology, Department of Internal Medicine, Kaohsiung Chang Gung Memorial Hospital and Chang Gung University College of Medicine, Kaohsiung, Taiwan, Republic of China; 5 Department of Biological Sciences, National Sun Yat-Sen University, Kaohsiung, Taiwan, Republic of China; 6 Institute of Biomedical Sciences, National Sun Yat-Sen University, Kaohsiung, Taiwan, Republic of China; 7 Department of Emergency Medicine, E-DA Hospital, I-Shou University, Kaohsiung, Taiwan, Republic of China; 8 Department of Biological Science and Technology, National Pingtung University of Science and Technology, Pingtung, Taiwan, Republic of China

## Abstract

**Background:**

We tested whether apoptotic adipose-derived mesenchymal stem cells (A-ADMSCs) were superior to healthy (H)-ADMSCs at attenuating organ damage and mortality in sepsis syndrome following cecal ligation and puncture (CLP).

**Methods:**

Adult male rats were categorized into group 1 (sham control), group 2 (CLP), group 3 [CLP + H-ADMSC administered 0.5, 6, and 18 h after CLP], group 4 [CLP + A-ADMSC administered as per group 3].

**Results:**

Circulating peak TNF-α level, at 6 h, was highest in groups 2 and 3, and higher in group 4 than group 1 (p < 0.0001). Immune reactivity (indicated by circulating and splenic helper-, cytoxic-, and regulatory-T cells) at 24 and 72 h exhibited the same pattern as TNF-α amongst the groups (all p < 0.0001). The mononuclear-cell early and late apoptosis level and organ damage parameters of liver (AST, ALT), kidney (creatinine) and lung (arterial oxygen saturation) also displayed a similar pattern to TNF-α levels (all p < 0.001). Protein levels of inflammatory (TNF-α, MMP-9, NF-κB, ICAM-1), oxidative (oxidized protein) and apoptotic (Bax, caspase-3, PARP) biomarkers were higher in groups 2 and 3 than group 1, whereas anti-apoptotic (Bcl-2) biomarker was lower in groups 2 and 3 than in group 1 but anti-oxidant (GR, GPx, HO-1, NQO-1) showed an opposite way of Bcl-2; these patterns were reversed for group 4 (all p < 0.001). Mortality was highest in group 3 and higher in group 2 than group 4 than group 1 (all p < 0.001).

**Conclusions:**

A-ADMSC therapy protected major organs from damage and improved prognosis in rats with sepsis syndrome.

## Introduction

Sepsis syndrome (i.e. systemic inflammatory response associated with infection) remains the leading cause of mortality in ICUs, ranging from 20% in sepsis to over 60% in septic shock [1-3], despite advancements in its management and in the understanding of its pathophysiology [1,2]. In the United States, it accounts for as many deaths annually as myocardial infarctions [1]. Clearly, there is an urgent need for innovative and efficacious therapies for its treatment.

The incidence of sepsis syndrome and its prognosis are clear [1-4], however its underlying mechanisms remain heavily debated [4-7]. Overwhelming inflammation is proposed to play a crucial role in the patient’s/host’s response to septic challenge [4,5]. This hyper-inflammatory response involves the innate immune system [5-8], neutrophil and macrophage accumulation [5], cytokines secretion [7,9], recruitment of T and B cells [5,10] and formation of antibodies [11] in an attempt to eliminate causative pathogens but this process also causes bystander attack on the major organs/tissues, leading to anergy of host-defense mechanisms, rapid organ failure and potential decline to death [12].

Previous studies [4-12] have addressed the hypothesis that uncontrolled immune reactivity may therefore be settled by new immunomodulatory therapeutics. Unfortunately, clinical trails of immunoglobulin therapy for patients with severe sepsis syndrome have yielded disappointing results [13]. However, recent studies have shown that mesenchymal stem cell (MSC) therapy can down-regulate innate and adaptive immunity [14]. One particularly intriguing finding was that apoptotic MSC possess intense anti-inflammatory and immunomodulatory properties [15].

Hence, MSC therapy for various inflammatory diseases/sepsis has been investigated [16-18] but reviewed results appear to be contradictory [19]. This may partly be explained by the heterogeneous nature of severe sepsis and also by the variation in tissues from which the MSCs have been isolated; one source may have a different capacity to immunoregulate than another. Interestingly, adipose tissue-derived (AD) MSCs may have more potent immunomodulatory capacity than bone marrow-derived MSCs [20]. Moreover, we have recently demonstrated that treatment with ADMSCs profoundly reduced rodent acute lung ischemia-reperfusion injury through highly significant suppression of oxidative stress and inflammation [21]. MSC therapy has reportedly been effective at reducing mortality from cecal ligation and puncture (CLP)-induced sepsis syndrome by rebalancing immune homeostasis and anti-inflammation, but such data remains very limited [18,22]. Such potentially promising therapeutic possibilities need further investigation before any translation towards clinical application.

Accordingly, using a rodent model of sepsis-syndrome induced by CLP, this study tested the hypothesis that 1) healthy (H)-ADMSC therapy might significantly reduce rat mortality and 2) apoptotic (A)-ADMSC therapy might be superior to H-ADMSC therapy at reducing rat mortality by attenuating the inflammatory response and immunomodulation. The second hypothesis was basic on the previous findings [15] and the findings of our recent experimental study which demonstrated that combined therapy with A-ADMSC and melatonin was superior to the combined therapy with H-ADMSC and melatonin for reducing acute rat lung ischemia-reperfusion injury [23].

## Materials and methods

### Ethics

All animal experimental procedures were approved by the Institute of Animal Care and Use Committee at our hospital and performed in accordance with the Guide for the Care and Use of Laboratory Animals (NIH publication No. 85-23, National Academy Press, Washington, DC, USA, revised 1996). All the technicians who performed the bench work were blinded to the treatment protocol.

### Animal groups and isolation of adipose tissue for culture of adipose-derived mesenchymal stem cells

Pathogen-free, adult male Sprague-Dawley (SD) rats weighing 400-450 g (Charles River Technology, BioLASCO Taiwan Co. Ltd., Taiwan) were randomized into sham procedure (SP) controls (cecal exposure *without* ligature and puncture), CLP + saline (3.0 cc, intra-peritoneally at 30 min, 6 h, and 18 h after CLP), CLP + H-ADMSC [(autologous 1.2 × 10^6^ cells) at 30 min, 6 h, and 18 h after CLP], CLP + A-ADMSC [(autologous 1.2 × 10^6^ cells) at 30 min, 6 h, and 18 h after CLP; n = 16 animals per group].

There were two reasons for why the ADMSCs were administered at 30 min, 6 h, and 18 h after CLP. First, we have recently demonstrated that ADMSC administration at the intervals of 30 min, 6 h, and 18 h after acute rat kidney ischemia-reperfusion (IR) injury by penile venous transfusion markedly attenuated acute IR-induced kidney injury [21]. Second, we proposed that the results of this preclinical study could provide useful information for our future clinical application. Accordingly, these intervals were initially chosen in an attempt to mimic the clinical scheduling of antibiotics for patients with sepsis syndrome.

Rats in groups CLP + H-ADMSC and CLP + A-ADMSC were anesthetized with inhalational 2.0% isoflurane 14 days before CLP in order to harvest autologous peri-epididymal adipose tissue as we recently reported [21]. Isolated ADMSCs were cultured in a 100 mm diameter dish with 10 mL DMEM culture medium containing 10% FBS for 14 days. Flow cytometric analysis was performed for identification of cellular characteristics (i.e., stem cell surface markers) after cell-labeling with appropriate antibodies on day 14 of cell cultivation prior to implantation (Table 1).

**Table 1:** Flow cytometric analysis of H-ADMSC and A-ADMSC surface markers following Day-14 cell culture

**Stem cell surface markers**	**H**-**ADMSC**	**A**-ADMSC*	**p**-**value**
CD31+	4.4 ± 1.9	3.1 ± 2.2	0.871
CD34+	12.6 ± 5.6	11.2 ± 5.9	0.729
VEGF+	29.2 ± 8.9	15.9 ± 5.7	0.027
CD45+	13.7 ± 4.3	6.0 ± 3.3	0.022
C-kit+	14.3 ± 4.9	8.9 ± 2.8	0.028
Sca-1+	1.9 ± 0.7	1.6 ± 0.9	0.454
CD133+	8.6 ± 4.2	5.7 ± 3.4	0.312
CD90+	92.4 ± 3.7	81.4 ± 2.9	0.029
CD271+	28.3 ± 4.2	10.1 ± 2.9	0.004
Early apoptosis (annexin V+/PI-)	1.03 ± 1.17	10.48 ± 3.67	<0.0001
Late apoptosis (annexin V+/PI+)	4.27 ± 0.59	18.18 ± 4.75	<0.0001

Data are expressed as %.

n = 8 in each experimental study.

H = healthy; A = apoptosis; ADMSC = adipose-derived mesenchymal stem cell; VEGF = vascular endothelial growth factor.

* An additional 96 hour serum-deprived cell culture was performed for A-ADMSCs.

### Cecal ligation and puncture (CLP), sham procedure (SP) and measurement of tail systolic blood pressure (SBP)

Rats were anesthetized with inhalational 2.0% isoflurane and placed supine on a warming pad at 37°C with the abdomen shaved. Under sterile conditions, the abdominal skin and muscle were opened and the cecum exposed in all groups. In the SP control animals, the abdomen was then closed and the animal allowed to recover from anesthesia. In the experimental CLP groups, the cecum was prolene suture ligated over its distal portion (i.e., distal ligation) and the cecum distal to the ligature was punctured twice with an 18# needle to allow the cecal contents to be expressed intraperitoneally, as previously described [24]. The wound was closed and the animal allowed to recover from anesthesia.

The tail SBP was measured (Kent Scientific Corporation, Model no: CODA, U.S.A) by a technician who was blinded to the treatment protocols prior to and at 9 h and 18 h after CLP or SP (CLP/SP).

### Estimation of SD Rat study group sizes

Based on previous work [24], we estimated that rat mortality at 72 h after CLP without treatment would be about 50%. Additionally, if clinically significant findings were to be encountered it was calculated that at least eight surviving rats would be required in any one group for statistical significance to be reached at 72 h after CLP. Accordingly, each group was assigned sixteen rats randomly. The time of death for each rat was recorded.

### Definition of healthy and apoptotic ADMSCs

Healthy ADMSCs were those cultured in normal culture medium with adequate nutrition. Serum deprivation of cells *in vitro* induces apoptosis [25]; hence, apoptotic ADMSCs were first cultured in normal culture medium followed by 96 hours of serum-free cell culture. The percentages of viable and apoptotic cells were determined by flow cytometry using double staining of annexin V and propidium iodide (PI); this is a simple and popular method for the identification of apoptotic cells (i.e. early [annexin V+/PI-] and late [annexin V+/PI+] phases of apoptosis).

### Isolation of splenocytes

Splenocytes were obtained by homogenization of the spleen using a Tenbroeck tissue grinder followed by passage through a 0.4-mm-pore-size cell strainer to obtain a single cell suspension. Splenocytes were then suspended in RPMI and speared by Ficoll-paque™ Plus (GE Healthcare).

### Flow cytometric quantification of helper T cells, cytotoxic T cells and regulatory T cells (tregs)

Peripheral blood mononuclear cells (PBMCs) were obtained from the tail vein using a 27# needle. PBMCs and splenocytes (1.0 × 10^6^ cells) were triple-stained with FITC-anti-CD3 (BioLegend), PE-anti-CD8a (BD Bioscience) and PE-Cy™5 anti-CD4(BD bioscience). To identify CD4^+^CD25^+^Foxp3^+^ Tregs, PBMCs and splenocytes were triple-stained with Alexa Fluor® 488-anti-CD25 (BioLegend), PE-anti-Foxp3 (BioLegend) and PE-Cy™5 anti-CD4 (BD bioscience) according to the manufacturer's protocol of Foxp3 Fix/Perm buffer set. The numbers of CD3^+^CD4^+^ helper T cells, CD3^+^CD8^+^ cytotoxic T cells and CD4^+^CD25^+^Foxp3^+^ Tregs were analyzed using flow cytometry (FC500, Beckman Coulter).

### Analyses of circulating biochemical markers

Blood samples were stored at -80 ^o^C until analyses of tumor necrosis factor (TNF)-α, blood urea nitrogen (BUN), creatinine, aspartate aminotransferase (AST), alanine aminotransferase (ALT) were performed in batches at the end of the experiment. Serum TNF-α concentration was assessed in duplicate with a commercially available ELISA kit (R&D systems, Inc. Minneapolis, MN). Intra-individual variability in TNF-α level was assessed in each group. The mean intra-assay coefficients of variance were all less than 4.0%. Circulating levels of BUN, creatinine, AST, ALT and white blood cell (WBC) count were measured at 24 h and 72 h after CLP/SP with standard laboratory methods.

### Western blot analysis of left ventricular specimens

Equal amounts (10-30 μg) of protein extracts from the left ventricle were loaded and separated by SDS-PAGE using 8-10% acrylamide gradients. Following electrophoresis, the separated proteins were transferred electrophoretically to a polyvinylidene difluoride (PVDF) membrane (Amersham Biosciences). Nonspecific proteins were blocked by incubating the membrane in blocking buffer (5% nonfat dry milk in T-TBS containing 0.05% Tween 20) overnight. The membranes were incubated with monoclonal antibodies against intercellular adhesion molecule (ICAM)-1 (1: 2000, Abcam), and polyclonal antibodies against TNF-α (1: 1000, Cell Signaling), nuclear factor (NF)-κB (1: 250, Abcam), platelet-derived growth factor (PDGF) (1:500, Abcam), matrix metalloproteinase (MMP)-9(1:5000, Abcam), glutathione peroxidase (GPx) (1:2000, Abcam), glutathione reductase (GR) (1:1000, Abcam), NAD(P)H quinone oxidoreductase (NQO) 1 (1: 1000, Abcam), heme oxygenase (HO)-1 (1: 250, Abcam), Bax (1: 1000, Abcam), caspase 3 (1: 1000, Cell Signaling), poly (ADP-ribose) polymerase (PARP) (1: 1000, Cell Signaling), Bcl-2 (1:250, Abcam) and cytochrome C (1: 2000, BD). Signals were detected with horseradish peroxidase (HRP)-conjugated goat anti-mouse, -rat, or -rabbit IgG.

The Oxyblot Oxidized Protein Detection Kit was purchased from Chemicon (S7150). The procedure of 2,4-dinitrophenylhydrazine (DNPH) derivatization was carried out on 6 μg of protein for 15 minutes according to the manufacturer’s instructions. One-dimensional electrophoresis was carried out on 12% SDS/polyacrylamide gel after DNPH derivatization. Proteins were transferred to nitrocellulose membranes which were then incubated in the primary antibody solution (anti-DNP 1: 150) for two hours, followed by incubation with the second antibody solution (1:300) for one hour at room temperature. The washing procedure was repeated eight times within 40 minutes.

Immunoreactive bands were visualized by enhanced chemiluminescence (ECL; Amersham Biosciences), which was then exposed to Biomax L film (Kodak). For quantification, ECL signals were digitized using Labwork software (UVP). For oxyblot protein analysis, a standard control was loaded on each gel.

### Statistical analyses

Quantitative data are expressed as mean ± SD. Statistical analysis was performed by ANOVA followed by Bonferroni multiple-comparison post hoc test. The survival was estimated using the Kaplan–Meier method, followed by comparing the statistical differences between various subgroups using the nonparametric linear log-rank test. All analyses were conducted using SAS statistical software for Windows version 8.2 (SAS institute, Cary, NC). A probability value <0.05 was considered statistically significant.

## Results

### Flow cytometric analysis of H-ADMSC and A-ADMSC surface markers following Day-14 cell culture

Flow cytometric analysis revealed that stem cell (CD45+, C-kit+, CD90+, CD271+) and vascular endothelial cell [vascular endothelial growth factor (VEGF)] surface markers were significantly lower in A-ADMSCs than in H-ADMSCs after 14-day cell culture (with the additional 96 h starvation for A-ADMSCs) (Table 1). However, the other stem cell (Sca-1+, CD133+) and endothelial progenitor cell (CD31+, CD34+) surface markers did not differ between these two groups (Table 1). Of importance, both early and late cellular apoptosis were significantly higher in A-ADMSCs than in H-ADMSCs.

### Rat mortality rate 72 hours after CLP

**Figure 1:**
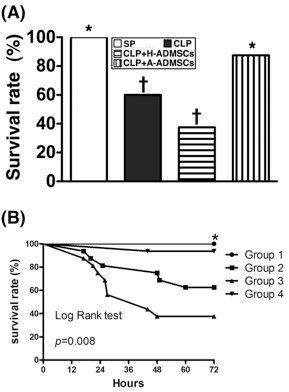
**Rat survival 72 h after cecal ligation and puncture(CLP)or sham procedure(SP).A**) By 72 h after the CLP procedure, the survival rate of groups CLP and CLP + H-ADMSC were significantly lower than in groups SP and CLP + A-ADMSC (all p values < 0.05) but it showed no difference between groups CLP and CLP + H-ADMSC (p = p = 0.289) or between groups SP and CLP + A-ADMSC (p = 1.0). * vs. other bars with different symbols, p < 0.001. All statistical analyses performed using one-way ANOVA followed by Bonferroni multiple comparison post hoc test. Symbols (*, †) indicating statistical significance (at 0.05 level). **B**) Kaplan-Meyer analysis comparing survival amongst the four groups, demonstrating that 72 h cumulative mortality was significantly higher in the CLP + H-ADMSC group and untreated CLP group than in SP controls and A-ADMSC group (p < 0.001). There were no significant survival differences between groups SP and CLP + A-ADMSC (p = 1.0), or between groups CLP and CLP + H-ADMSC (p = 0.289). H-ADMSCs = healthy adipose-derived mesenchymal stem cells; A-ADMSCs = apoptotic adipose-derived mesenchymal stem cells.

Unexpectedly, the mortality rate was notably higher in CLP animals treated with H-ADMSCs [mortality 62.5% (10/16)] than CLP animals without treatment [mortality 37.5% (6/16)] but this difference did not reach statistical significance (p = 0.289) (Figure 1A). Mortality rate in CLP animals treated with A-ADMSC [mortality 6.25% (1/16)] and in the SP control (mortality 0%) was similar (Figure 1B). Interestingly, the highest incidence of deaths in groups 2 and 3 occurred within 16 h and 24 h after CLP. Of importance, mortality was significantly higher in groups CLP and CLP + H-ADMSC than in groups SP and CLP + A-ADMSC (p < 0.04).

### Systolic blood pressure at 0, 9 and 18 hours after CLP/SP

**Figure 2:**
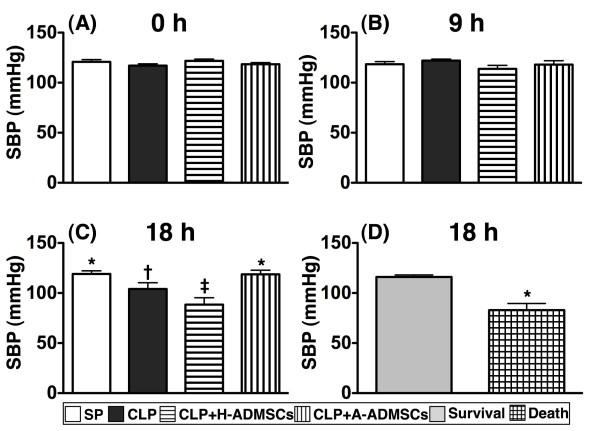
**Time course of systolic blood pressure** (**SBP**) **among the animals prior to and after cecal ligation and puncture(CLP)or sham procedure(SP).A**) and **B**) The SBP at 0 h and at 9 h after CLP/SP were similar amongst the four groups. **C**) Illustrating the SBP at 24 h. * vs. other bars with different symbols, p < 0.001. **D**) Comparing the SBP at 24 hour after CLP/SP between the animals that ultimately survived and the animals that ultimately died. * vs. survival group, p < 0.0001. All statistical analyses utilized one-way ANOVA, followed by Bonferroni multiple comparison post hoc test. Symbols (*, †, ‡) indicating significance (at 0.05 level). CLP = cecal ligation and puncture; Normal indicates sham procedure; H-ADMSCs = healthy adipose-derived mesenchymal stem cells; A-ADMSCs = apoptotic adipose-derived mesenchymal stem cells.

The SBP was similar amongst animals in the four groups at 0 h and at 9 h after CLP/SP (Figure 2, A and B). By 18 h, however, SBP was significantly lower in CLP + H-ADMSC group than in the other groups, significantly lower in CLP group than in groups SP and CLP + A-ADMSC, and similar in groups SP and CLP + A-ADMSC (Figure 2C). Additionally, the SBP at 18 h was significantly lower amongst the animals that ultimately died compared with those that ultimately survived (Figure 2D).

### Laboratory findings at 24 h and 72 h after CLP/SP

By 24 h after CLP/SP, serum ALT, BUN, creatinine and WBC count did not differ between groups (Table 2). However, serum AST was significantly higher in groups CLP and CLP + H-ADMSC than in groups SP and CLP + A-ADMSC, significantly higher in group CLP + A-ADMSC than in group SP, and not significantly different between groups CLP and CLP + H-ADMSC (Table 2). At 72 h, serum ALT remained similar between the four groups. However, AST level was significantly higher in group CLP + H-ADMSC than the other groups, significantly higher in group CLP than in groups SP and CLP + A-ADMSC, and significantly higher in group CLP + A-ADMSC than in group SP (Table 2). BUN levels were similar in groups CLP, CLP + H-ADMSC and CLP + A-ADMSC, but significantly lower in group SP than the other groups. Creatinine was significantly higher in groups CLP and CLP + H-ADMSC than in groups SP and CLP + A-ADMSC but it did not differ between groups CLP and CLP + H-ADMSC or between groups SP and CLP + A-ADMSC. The WBC count was significantly highest in group CLP, significantly higher in groups CLP + H-ADMSC and CLP + A-ADMSC than in group SP, and not significantly different in groups CLP + H-ADMSC and CLP + A-ADMSC (Table 2).

**Table 2:** Hematologic and biochemical studies among the four groups

**Variables**	**SP**	**CLP**	**CLP** + **H**-**ADMSC**	**CLP** + **A**-**ADMSC**	**p**-**value**
At 24 hour					
AST	57 ± 7.1^a^	153.7 ± 14.1^b^	160.9 ± 27.5^b^	126.1 ± 20.9^c^	<0.001
ALT	31.9 ± 6.7	46.1 ± 5.1	46.9 ± 18.8	35.3 ± 11.5	0.06
BUN	20.3 ± 2.4	21.8 ± 6.9	22.1 ± 6.3	23.4 ± 9.3	0.849
Creatinine	0.51 ± 0.12	0.77 ± 0.15	0.74 ± 0.25	0.66 ± 0.14	0.051
WBC count	8.1 ± 2.0	4.9 ± 2.6	7.7 ± 2.5	6.8 ± 1.4	0.057
At 72 hour					
AST	46.1 ± 11.1^a^	106.6 ± 12.4^b^	134 ± 23.4^c^	81.6 ± 17.1^d^	<0.001
ALT	25 ± 5.1	34.6 ± 10.1	36 ± 7.6	24.7 ± 14	0.06
BUN	16.7 ± 1.9^a^	28.8 ± 6.5^b^	28.3 ± 14.2^b^	25 ± 5.9^b^	0.018
Creatinine	0.5 ± 0.07^a^	0.78 ± 0.14^b^	0.83 ± 0.17^b^	0.61 ± 0.11^a^	<0.001
WBC count	7.7 ± 1.9^a^	12.6 ± 2.8^b^	9.2 ± 3.1^c^	9.5 ± 2.3^c^	0.016

Data are expressed as means ± SD.

AST = aspartate aminotransferase; ALT = alanine aminotransferase; BUN = blood urea nitrogen; WBC = white blood cell count.

SP = sham procedure; CLP = cecal ligation and puncture; H-ADMSC = healthy adipose-derived mesenchymal stem cells; A-ADMSCs = apoptotic adipose-derived mesenchymal stem cells.

### Serial changes of circulating tumor necrosis factor-alpha (TNF-α) levels after CLP/SP

**Figure 3:**
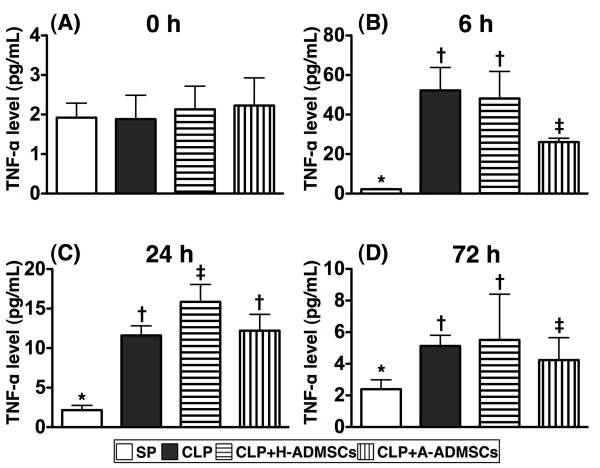
**Serial changes of circulating levels of tumor necrotic factor(TNF)-α.A**) Circulating TNF-α levels did not differ amongst the four groups prior to CLP/SP. **B**) Circulating TNF-α levels amongst the four groups at 6 h after CLP/SP. * vs. other bars with different symbols, p < 0.0001. **C**) Circulating TNF-α levels amongst the four groups at 24 h after CLP/SP. * vs. other bars with different symbols, p < 0.0001. **D**) Circulating TNF-α levels amongst the four groups at 72 h after CLP/SP. * vs. other bars with different symbols, p < 0.001. All statistical analyses utilized one-way ANOVA, followed by Bonferroni multiple comparison post hoc test. Symbols (*, †, ‡) indicating significance (at 0.05 level). CLP = cecal ligation and puncture; Normal indicates sham procedure; H-ADMSCs = healthy adipose-derived mesenchymal stem cells; A-ADMSCs = apoptotic adipose-derived mesenchymal stem cells.

Levels of serum TNF-α, an index of acute inflammation, were similar amongst the groups before CLP/SP (Figure 3A). By 6 h after CLP/SP, however, serum TNF-α was significantly higher in groups CLP and CLP + H-ADMSC than in groups SP and CLP + A-ADMSC, significantly higher in group CLP + A-ADMSC than in group SP, and not significantly different between groups CLP and CLP + H-ADMSC (Figure 3B). By 24 h, TNF-α was significantly higher in group CLP + H-ADMSC than in the other groups, significantly higher in groups CLP and CLP + A-ADMSC than in group SP, and not statistically different in groups CLP and CLP + A-ADMSC (Figure 3C). By 72 h, the differences in this parameter between groups were as they were at the 6 h timepoint, although the actual serum levels were generally reduced in groups CLP, CLP + H-ADMSC and CLP + A-ADMSC (Figure 3D).

### The immune reactivity at circulation and spleen at 72 h after CLP/SP procedure

**Figure 4:**
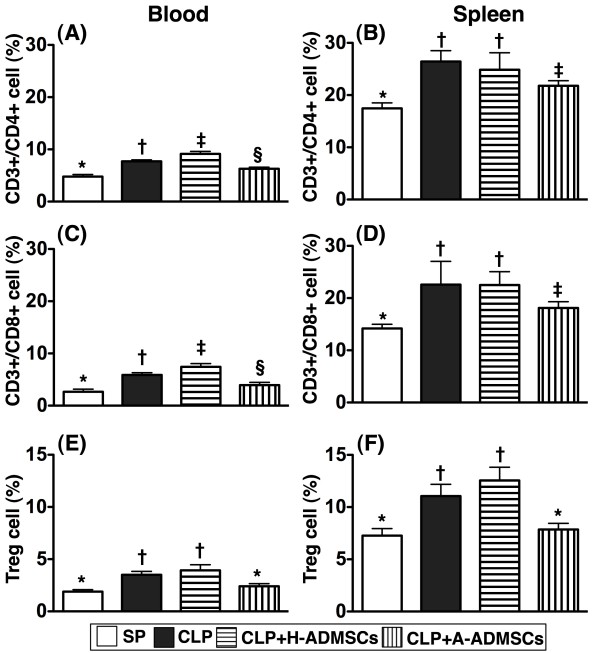
**Circulating and splenic levels of T lymphocytes at 72 h after CLP/SP.A**) Circulating levels of CD3+/CD4+ cells (helper T cells). * vs. other bars with different symbols, p < 0.001. **B**) Splenic levels of CD3+/CD4+ cells. * vs. other bars with different symbols, p < 0.001. **C**) Circulating levels of CD3+/CD8+ cells (cytotoxic T cells). * vs. other bars with different symbols, p < 0.001. **D**) Splenic levels of CD3+/CD8+ cells. * vs. other bars with different symbols, p < 0.001. **E**) Circulating levels of CD4 + CD25 + Foxp3+ cells (Treg cells). * vs. other bars with different symbols, p < 0.001. **F**) Splenic levels of Treg cells. * vs. other bars with different symbols, p < 0.001. All statistical analyses used one-way ANOVA, followed by Bonferroni multiple comparison post hoc test. Symbols (*, †, ‡, §) indicating significance (at 0.05 level). CLP = cecal ligation and puncture; Normal indicates sham procedure; H-ADMSCs = healthy adipose-derived mesenchymal stem cells; A-ADMSCs = apoptotic adipose-derived mesenchymal stem cells.

To assess the acute immune response, circulating and splenic levels of CD3+/CD4+ helper T cells, CD3+/CD8+ cytotoxic T cells and CD4 + CD25 + Foxp3+ Tregs were measured 72 h after CLP/SP. Surprisingly, there were significantly higher circulating levels of helper and cytotoxic T cells in group CLP + H-ADMSC than in the other groups, significantly higher levels in group CLP than in groups SP and CLP + A-ADMSC, and significantly higher levels in group CLP + A-ADMSC than in group SP (Figure 4, A and C). Levels of helper and cytotoxic T cells in spleen were higher in groups CLP and CLP + H-ADMSC than in groups SP and CLP + A-ADMSC, higher in group CLP + H-ADMSC than in group SP, and similar in groups CLP and CLP + H-ADMSC (Figure 4B and D). Furthermore, the circulating and splenic levels of Tregs, an index of immune down-regulation, were significantly higher in groups CLP and CLP + H-ADMSC than in groups SP and CLP + A-ADMSC; levels between groups CLP and CLP + H-ADMSC were not significantly different, nor were they statistically different between groups SP and CLP + A-ADMSC (Figure 4E and F). Of importance, the levels of these biomarkers were significantly lower in the peripheral circulation than in spleen.

### Early or late mononuclear cell (MNC) apoptosis in the circulation at 72 h after CLP/SP

**Figure 5:**
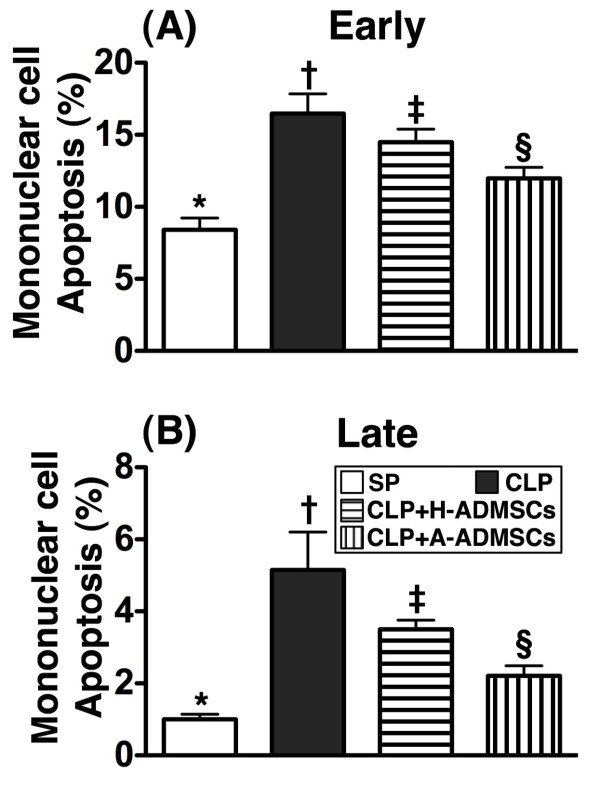
**Circulating level of mononuclear cell apoptosis at 72 h after the CLP/SP.A**) Early apoptosis. * vs. other bars with different symbols, p < 0.0001. **B**) Late apoptosis. * vs. other bars with different symbols, p < 0.0001. All statistical analyses used one-way ANOVA, followed by Bonferroni multiple comparison post hoc test. Symbols (*, †, ‡, §) indicating significance (at 0.05 level). CLP = cecal ligation and puncture; Normal indicates sham procedure; H-ADMSCs = healthy adipose-derived mesenchymal stem cells; A-ADMSCs = apoptotic adipose-derived mesenchymal stem cells.

Flow cytometry demonstrated that early apoptosis of circulating MNCs was highest in group CLP, significantly higher in group CLP + H-ADMSC than in groups SP and CLP + A-ADMSC and significantly higher in group CLP + A-ADMSC than in group SP (Figure 5A). Late apoptosis of MNCs in the peripheral circulation exhibited an identical pattern, although the incidence of late apoptosis was lower than that of early apoptosis (Figure 5B).

### The ratio of heart, lung and kidney weight to body weight (BW) and arterial oxygen saturation (O2 sat, %) 72 hrs after CLP/SP

**Figure 6:**
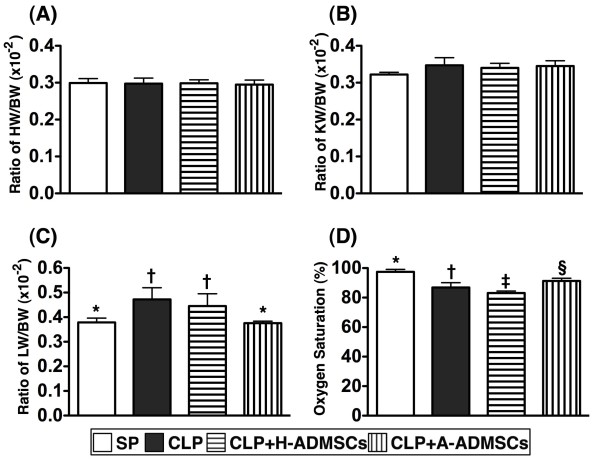
**Ratio of heart weight(HW),kidney weight(KW)and lung weight(LW)to the body weight(BW)at 72 h after CLP/SP.A**) The ratio of HW to BW at 72 h after CLP/SP did not differ between the four groups. **B**) The ratio of KW to BW at 72 h after CLP/SP did not differ between the four groups. **C**) The ratio of LW to BW at 72 h after CLP/SP, respectively. * vs. other bars with different symbols, p < 0.001. **D**) The arterial oxygen saturation (%) at 72 h after CLP/SP. * vs. other bars with different symbols, p < 0.001. All statistical analyses used one-way ANOVA, followed by Bonferroni multiple comparison post hoc test. Symbols (*, †, ‡, §) indicating significance (at 0.05 level). CLP = cecal ligation and puncture; Normal indicates sham procedure; H-ADMSCs = healthy adipose-derived mesenchymal stem cells; A-ADMSCs = apoptotic adipose-derived mesenchymal stem cells.

The ratio of heart weight to BW (Figure 6A) and kidney weight to BW (Figure 6B) at 72 h was similar amongst the four groups. The ratio of lung weight to BW, however, was significantly higher in groups CLP and CLP + H-ADMSC than in groups SP and CLP + A-ADMSC at 72 h after CLP/SP (Figure 6C); this parameter did not differ between groups CLP and CLP + H-ADMSC or between groups SP and CLP + A-ADMSC.

Arterial O2 sat (%) was lower in group CLP + H-ADMSC than in the other groups, significantly lower in group CLP than in groups SP and CLP + A-ADMSC, and significantly lower in group CLP + A-ADMSC than in group SP at 72 h after CLP/SP (Figure 6D).

### Western blot analyses of inflammatory biomarkers in left ventricular (LV) myocardium 72 h after CLP/SP

**Figure 7:**
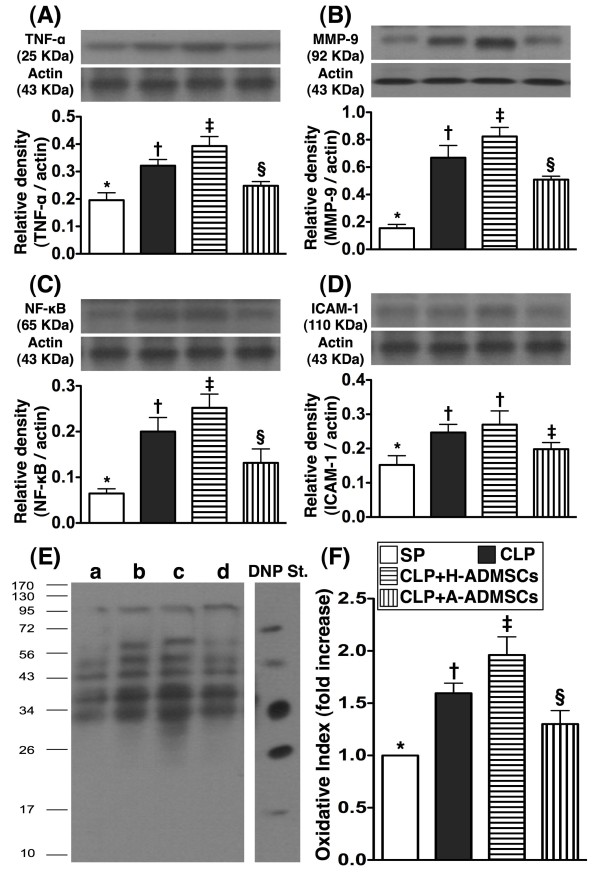
**Protein expressions of inflammatory and oxidative stress biomarkers of left ventricular myocardium at 72 h after CLP/SP.A**, **B** and **C**) The protein expressions of tumor necrotic factor (TNF)-α (**A**), matrix metalloproteinase (MMP)-9 (**B**) and nuclear factor (NF)-κB (**C**) exhibited an identical pattern amongst the four groups. * vs. other bars with different symbols, p < 0.0001. **D**) The protein expression of intercellular adhesion molecule (ICAM)-1. * vs. other bars with different symbols, p < 0.0001. **E** &**F**) Expression of protein carbonyls (i.e., oxidized protein), an oxidative index, in the four groups of animals [Note: Right lane and left lane shown on left lower panel (**E**) representing control oxidized molecular protein standard and protein molecular weight marker, respectively]. DNP = 1-3 dinitrophenylhydrazone. * vs. other bars with different symbols, p < 0.0001. a = SP control; b = CLP; c = CLP + H-ADMSCs; d = CLP + A-ADMSCs. All statistical analyses used one-way ANOVA, followed by Bonferroni multiple comparison post hoc test. Symbols (*, †, ‡, §) indicating significance (at 0.05 level). CLP = cecal ligation and puncture; Normal indicates sham procedure; H-ADMSCs = healthy adipose-derived mesenchymal stem cells; A-ADMSCs = apoptotic adipose-derived mesenchymal stem cells.

The protein expressions of TNF-α, MMP-9, NF-κB, three indices of inflammation, and oxidized protein, an indicator of oxidative stress, were significantly higher in group CLP + H-ADMSC than in groups SP, CLP and CLP + A-ADMSC, significantly higher in group CLP than in groups SP and CLP + A-ADMSC, and significantly higher in group CLP + A-ADMSC than in group SP (Figure 7A to C, E and F). Protein expression of ICAM-1, another inflammatory biomarker, was significantly higher in groups CLP and CLP + A-ADMSC than in groups SP and CLP + A-ADMSC, and significantly higher in group CLP + A-ADMSC than in group SP; its expression in groups CLP and CLP + H-ADMSC was similar (Figure 7D).

### Western blot results of apoptotic biomarkers in left ventricular (LV) myocardium at 72 h after CLP/SP

**Figure 8:**
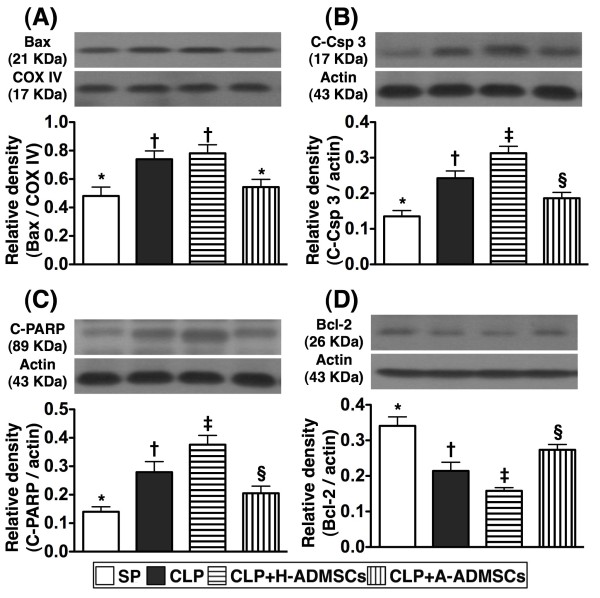
**Protein expressions of apoptotic and anti**-**apoptotic biomarkers of left ventricular myocardium at 72 h after CLP/SP.A**, **B** and **C**) The protein expressions of mitochondrial Bax (**A**), cleaved casapse 3 (C-Csp 3) (**B**) and cleaved poly (ADP-ribose) polymerase (C-PARP) exhibited a similar pattern amongst the four groups. * vs. other bars with different symbols, p < 0.001. **D**) The protein expression of Bcl-2. * vs. other bars with different symbols, p < 0.001. All statistical analyses used one-way ANOVA, followed by Bonferroni multiple comparison post hoc test. Symbols (*, †, ‡, §) indicating significance (at 0.05 level). CLP = cecal ligation and puncture; Normal indicates sham procedure; H-ADMSCs = healthy adipose-derived mesenchymal stem cells; A-ADMSCs = apoptotic adipose-derived mesenchymal stem cells.

Protein expression of mitochondrial Bax (i.e., active form), an indicator of cellular apoptosis, was significantly higher in groups CLP and CLP + H-ADMSC than in groups SP and CLP + A-ADMSC; no statistical differences were noted between groups CLP and CLP + H-ADMSC or between groups SP and CLP + A-ADMSC (Figure 8A). Additionally, protein expressions of cleaved (active) caspase 3 and PARP, two other indicators of cellular apoptosis, were highest in group CLP + H-ADMSC, significantly higher in group CLP than in groups SP and CLP + A-ADMSC, and significantly higher in group CLP + A-ADMSC than in group SP (Figure 8B and C). Conversely, protein expression of Bcl-2, an indicator of anti-apoptosis, was lowest in group CLP + H-ADMSC, significantly lower in group CLP than in groups SP and CLP + A-ADMSC, and significantly lower in group CLP + A-ADMSC than in group SP (Figure 8D).

### Western blot results of anti-oxidant and mitochondrial-preservation biomarkers in left ventricular (LV) myocardium at 72 h after CLP/SP

**Figure 9:**
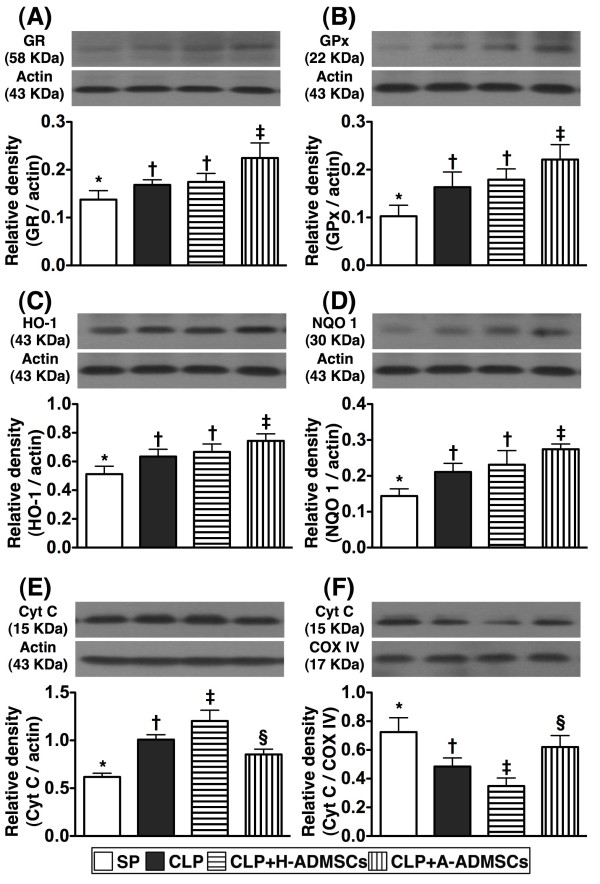
**Protein expressions of anti**-**oxidant and mitochondrial preservation biomarkers of left ventricular myocardium at 72 h after CLP/SP.A**, **B**, **C** and **D**) The protein expressions of glutathione reductase (GR) (**A**) glutathione peroxidase (GPx) (**B**), heme oxygenase (HO)-1 (**C**) and NAD(P)H quinone oxidoreductase (NQO) 1 (**D**) displayed an identical pattern amongst the four groups. * vs. other bars with different symbols, p < 0.001. **E** &**F**) Protein expression of cytosolic (**E**) and mitochondrial (**F**) cytochrome C (Cyt C). * vs. other bars with different symbols, p < 0.001. All statistical analyses used one-way ANOVA, followed by Bonferroni multiple comparison post hoc test. Symbols (*, †, ‡, §) indicating significance (at 0.05 level). CLP = cecal ligation and puncture; Normal indicates sham control; H-ADMSCs = healthy adipose-derived mesenchymal stem cells; A-ADMSCs = apoptotic adipose-derived mesenchymal stem cells.

Protein expressions of GR, GPx, HO-1, and NQO 1, four indices of anti-oxidants, were significantly higher in group CLP + A-ADMSC than the other groups, significantly higher in groups CLP and CLP + H-ADMSC than in group SP, and statistically similar in groups CLP and CLP + H-ADMSC (Figure 9A-D). Protein expression of cytochrome C at mitochondrial level, an indicator of mitochondrial preservation in LV myocardium, was significantly higher in group SP than the other groups, significantly higher in group CLP + A-ADMSC than groups CLP and CLP + H-ADMSC, and significantly higher in group CLP than in group CLP + H-ADMSC (Figure 9F). However, the expression of this biomarker at cytosolic level, an index of mitochondrial damage, exhibited a reversed pattern (Figure 9E).

## Discussion

This study, which investigated the impact of AMDSC administrations at three time intervals (i.e. at 0.5, 6 and 18 h) on sepsis syndrome, yielded several striking observations. First, unexpectedly, the circulating levels of TNF-α were notably higher in animals with H-ADMSC treatment than in animals without H-ADMSC treatment at 24 h and 72 h after CLP. However, at the same time intervals, the levels of this biomarker were remarkably reversed in those animals after A-ADMSC treatment. Second, surprisingly, the numbers of helper T cells, cytotoxic T cells and Tregs in both peripheral blood and spleen were identically increased in animals with and without H-ADSMC therapy and yet were significantly reduced after A-ADMSC therapy. Third, more surprisingly, mortality rate was relatively higher in animals receiving H-ADMSC therapy than in animals without H-ADMSC therapy. However, mortality was markedly reduced in animals after A-ADMSC therapy. These findings implicate that A-ADMSCs represent an endogenous therapeutic strategy that may be possible to enhance for maximum clinical benefit.

Many studies have shown that MSCs display multiple beneficial properties through their capacity for homing [26,27], angiogenesis, attenuating the inflammatory response [21,27], modulating immune cells [14,15,19], and promoting tissue regeneration [21,27]. Additionally, studies have also demonstrated that MSCs may be useful therapeutic adjuncts for reducing mortality in experimental models of sepsis syndrome by inhibiting the inflammatory and immunoregulatory response [18,22]. Surprisingly, the current study revealed that the mortality rate was notably higher in CLP animals that received H-ADMSC therapy than in CLP animals without treatment, which does not support the findings of others [18,22]. Conversely, however, mortality following CLP was significantly reduced by A-ADMSC therapy. In this way, our findings corroborated those of previous studies [18,22]. Our observations may, therefore, suggest that the immunomodulatory capacity and anti-inflammatory property that is more potent in A-ADMSCs than in H-ADMSCs played the crucial role for reducing sepsis-induced mortality. Our suggestion is supported by our following findings. Firstly, circulating TNF-α level was identically increased at the 6 h timepoint after CLP in H-ADMSC treated and untreated animals, becoming significantly increased in H-ADMSC treated animals at 24 and 72 h compared with untreated animals. However, the circulating TNF-α levels were significantly reversed after A-ADMSC treatment. Secondly, compared with SP controls, the circulating and splenic levels of helper T cells, cytotoxic T cells and Tregs were significantly higher in untreated CLP animals than in animals with A-ADMSC therapy at 72 h after CLP, and yet these levels were even higher in group CLP + H-ADMSC where animals were treated with H-ADMSC following CLP. These findings might, at least in part, support the findings that the mortality rate was notably higher in animals with H-ADMSC treatment than in those animals without treatment. These findings could also explain why the mortality rate was remarkably reduced in animals with A-ADMSC therapy than in animals with no treatment or with H-ADMSC therapy.

Major organs are frequently damaged by sepsis syndrome [1-4]. Consistent with previous reports [1-4], our results demonstrated that liver and renal functions were significantly impaired in CLP animals compared with SP controls. Additionally, arterial oxygen saturation, an index of lung function, was significantly lower whilst lung weight to body weight ratio was significantly increased (an index of lung damage) in CLP animals than in SP controls. Furthermore, of particular importance was that these functional organ damages were more severe in animals receiving H-ADMSC therapy than in those who underwent CLP without treatment. However, these functional damages were notably reversed after A-ADMSC therapy. Collectively, these findings could also partially explain why mortality was higher in the CLP group that was treated with H-ADMSC than in the untreated CLP group, which was remarkably reversed in the A-ADMSC treated CLP group. Recently, studies have shown that MSC therapy attenuated organ injury induced by endotoxemia [16,28]. In this way, the results of A-ADMSC therapy were consistent with these recent studies [16,28].

Apart from evaluating the impact of ADMSC therapy on protecting pulmonary and renal function, the current study also considered the molecular-cellular levels in left ventricular myocardium at 72 h after CLP/SP. Anatomically, the ratio of heart weight to body weight did not differ among the four groups. However, the protein expressions of inflammatory markers and oxidative stress were substantially higher in animals with than in animals without H-ADMSC therapy. These findings imply that H-ADMSC therapy did not enhance anti-inflammation either in the circulation or in the specific tissues/major organs in setting of sepsis syndrome. In contrast, A-ADMSC therapy markedly reduced the protein expressions of these inflammatory biomarkers. One recent study has also revealed that MSC therapy significantly reduced endotoxin elicited inflammatory biomarkers in left ventricular myocardium [16]. Our findings of A-ADMSC treatment therefore corroborate those of others [16] but this is also reinforced by our peripheral circulating measurements that demonstrated A-ADMSC therapy significantly enhanced anti-inflammation in sepsis syndrome. Besides, the protein expressions of antioxidants and mitochondrial preservation were remarkably lower in animals with and without H-ADMSC treatment compared with those animals that received A-ADMSC treatment. These findings highlight that A-ADMSC therapy not only enhanced anti-inflammatory capability but also enriched the anti-oxidant ability in the setting of sepsis syndrome.

Sepsis induced apoptosis has been well recognized in previous studies [28]. In the current study, one essential finding was that the number of circulating apoptotic mononuclear cells (i.e., early and late apoptosis) was substantially increased at 72 h in animals with or without H-ADMSC therapy after CLP than in SP controls. However, these biomarkers were substantially decreased in animals after A-ADMSC treatment. Similarly, the protein expressions of apoptotic biomarkers in left ventricular myocardium exhibited an identical pattern of circulating mononuclear cell apoptosis amongst the four groups 72 h after CLP/SP. Our findings, in addition to strengthening the findings of others [28], suggest that A-ADMSC therapy significantly prevented cellular apoptosis and ultimately protected the organ/tissue from sepsis-induced damage.

We remain uncertain why there was a discrepancy between our results of H-ADMSC therapy (ineffective in reducing mortality) and A-ADMSC therapy (effective in reducing mortality) and the findings of others in the setting of sepsis with MSC therapy (reportedly effective in reducing mortality) [18,22]. We propose that the following reasons could explain the discrepancy. First, we suggest that three dosages of H-ADMSC, especially the 3^rd^ dosage, might elicit a hyper-reactive immune response that mimicked a delayed hypersensitivity rather than immune desensitization. This suggestion is supported by our findings that the numbers of helper T cells, cytotoxic T cells and Tregs were substantially increased in both circulating and spleen levels at 72 h after CLP. This may lend some explanation to why the majority of the rats died more than 18 h after CLP. Second, previous studies have shown that more that 5%-25% of the prepared MSCs exhibited cellular apoptosis before they were injected or implanted into an ischemic area [15]. A further proportion of healthy injected living cells are susceptible to apoptosis within ischemic organ/tissue due to exposure to various proapoptotic or cytotoxic factors in an ischemic environment [15,29]. Apoptotic rather than healthy stem cells have been emphasized to possess a unique property of immune dampening by down-regulating innate and adaptive immunity, deactivating macrophages and dendritic cells, and stimulating regulatory T cells [15]. Our flow cytometric analyses showed that early apoptosis was less than 1.1% and late apoptosis was less than 4.5% in H-ADMSCs. However, more than 10% of early apoptosis and more than 18% of late apoptosis were found in A-ADMSCs after 96 h serum-deprived cell culture. These may explain the differing immunologic homeostasis, tolerance and mortality between animals with H-ADMSC therapy and animals with A-ADMSC therapy in the present study.

### Study limitations

This study is limited by the following factors. First, although the numbers of *in vitro* apoptosis of both H-ADMSCs and A-ADMSCs were estimated, we did not measure the time course of circulating numbers of apoptotic H-ADMSCs or A-ADMSCs. Therefore, we could not provide the incidence of apoptosis *in vivo* for these stem cells. Second, the study period was only 72 h, therefore we have not yet provided the long-term results of animals after ADMSC therapy in the setting of sepsis syndrome. Third, the study did not extensively surveyed the serial changes of circulating inflammatory cytokines/chemokines that might provide more useful information for predicting the prognostic outcome after sepsis syndrome. Finally, the H-ADMSCs were retained in 3 cc fetal bovine serum whereas the A-ADMSCs were retained in 3 cc serum-free culture medium prior to treat the animals. We remain uncertain if the fetal bovine serum also played some certain role on eliciting the immune response during the treatment course.

## Conclusions

A-ADMSC therapy appears superior to H-ADMSC therapy for preserving organ function and reducing mortality in rat sepsis syndrome. This indicates a potentially important role for A-ADMSC therapy for sepsis syndrome.

## Competing interests

The authors declare that they have no competing interests.

## Authors’ contributions

All authors have read and approved the final manuscript. CLC (Chang), SL, YYZ, JJS, and CKS designed the experiment, drafted and performed animal experiments. AC, HCS, THT, HTC, and SYC were responsible for the laboratory assay and troubleshooting. SL, CKS, and HKY participated in refinement of experiment protocol and coordination and helped in drafting the manuscript.
